# DOES AGE AFFECT CREATIVE PROCESS AND STYLE? A COMPARISON OF OLDER AND YOUNGER VISUAL ARTISTS

**DOI:** 10.1093/geroni/igz038.1558

**Published:** 2019-11-08

**Authors:** Monika Ardelt, Lucinda Orwoll

**Affiliations:** 1 University of Florida, Gainesville, Florida, United States; 2 Private Practice, Ann Arbor, Michigan, United States

## Abstract

This study investigated differences in the creative process and style between 85 older (age 60-89 years, M=72.39) and 63 younger (age 27-58 years, M=41.95) visual artists who were nominated as artistically creative exemplars. Answers to open-ended survey questions were coded and compared by age group. Results of t-tests showed that the described creative process and style were similar for older and younger artists, with many being inspired by their environment or ideas and engaging in an intuitive and visual style. However, younger artists were more likely than older artists to be inspired by ideas, words, life, and the work process and to use an intuitive, expressionistic, eclectic, spiritual, and textured style. Interestingly, younger artists were more likely to believe that older artists have greater artistic experience, maturity, willingness to take risks, and understanding of the art world, whereas older artists tended to think that young artists are more original.

